# Effectiveness of Leg Raise and Leg Fold Maneuver to Prevent Syncope During Extraction of Teeth: A Pilot Study

**DOI:** 10.7759/cureus.34488

**Published:** 2023-02-01

**Authors:** James Antony Bhagat M, Sakthi S, Nathiya B, Durairaj D, Thennarasu A.R.

**Affiliations:** 1 Department of Oral and Maxillofacial Surgery, Adhiparasakthi Dental College and Hospital, Melmaruvathur, IND

**Keywords:** extraction of teeth, anxiety during exodontia, vasovagal syncope, leg raise and leg fold maneuver, syncope, exodontia, prevention of syncope, syncope during extraction

## Abstract

Objectives

The primary objective of this study is to evaluate the effectiveness of leg raise and leg fold maneuvers to prevent syncope during extraction procedures.

Methods

This study included 30 patients with a previous history of syncope and dental anxiety. Patients were randomly divided into two groups consisting of 15 patients each. Group I (test group) patients were educated about a few physical maneuvers, and instructions were given preoperatively about when to perform them. Group II (control group) underwent extraction conventionally. The blood pressure, saturation, pulse rate, and clinical signs and symptoms of the patients were assessed preoperatively, intraoperatively, and postoperatively. Informed consent was obtained from all the patients.

Results

There is a significant difference between the control and study groups in terms of the occurrence of syncope and patient comfort. This indicates that the leg raise and leg fold maneuvers reduce the occurrence of syncope during extraction. No participant in the test group experienced syncope post-treatment, while five subjects (33.3%) experienced syncope in the control group.

Conclusion

Physical counterpressure maneuvers are a risk-free, effective, and low-cost treatment method in patients with vasovagal syncope. Leg raise and leg fold maneuvers improved the hemodynamics of the patients.

## Introduction

Oral surgeons and dentists need to be equipped with adequate training and knowledge to manage medical emergencies. The most commonly reported medical emergency in dental practice is syncope, which accounts for at least half of all medical emergencies [1]. Syncope is a symptom characterized by a transient loss of both consciousness and postural tone. An episode occurs rapidly, and the patient recovers quickly. Vasovagal syncope (VVS), also known as "common faint," is a neurally mediated syndrome associated with hypotension and relative bradycardia due to cerebral hypoperfusion [2]. The incidence of vasovagal syncope in a dental hospital is around 2% [3]. Young males are more commonly affected than females [4]. Literature other than dental specialties suggests that women are more prone to syncope [5-10]. Vasovagal syncope may occur in every age group. A bimodal age distribution with a peak incidence at the ages of 20-29 years and 70-79 years is seen [11]. Typical syncope features can be obtained from the patient’s own words.

In Belgium, 34.3% of dentists have encountered a vasovagal episode during their careers [12], although the majority of dentists and oral surgeons would have come across syncope in their careers [13,14]. The frequent occurrence in dental practices may be partially explained by psychogenic factors such as dental fear, which induces emotional stress and pain, fear of visiting the dentist, and the dental setting or treatment that affects 10%-15% of the population [15-17]. Oral surgical procedures, including the use of local anesthesia, can be emotionally challenging from a patient’s perspective [18]. Traditionally, syncope during extraction is treated by placing the patient in a supine position with feet elevated at 10° and maintaining an open airway to re-establish cerebral perfusion [19]. The main objective of this article is to provide an overview of the management of vasovagal syncope in patients with a previous history of syncope and anxiety using simple physical maneuvers that can be practiced during extraction to reduce the development of vasovagal syncope.

## Materials and methods

A pilot study was designed since we have advocated a novel method to prevent syncope during extraction. This pilot study was designed with a total of 30 patients. This study included only patients with a previous history of syncope and dental anxiety. Patients were divided into two groups consisting of 15 patients each. The study group consisted of 15 patients who were taught leg raise and leg fold maneuvers and were asked to perform the maneuver at regular intervals during extraction, and the control group consisted of 15 patients who underwent extraction in the conventional method.

Inclusion criteria

This study includes subjects ranging from 18-75 years of age with a previous history of syncope and dental anxiety.

Exclusion criteria

Patients with musculoskeletal disease; patients with suspected or overt heart disease with a high likelihood of cardiac syncope; patients with orthostatic hypotension; patients with episodes of loss of consciousness different from syncope; patients who are psychologically, physically, or cognitively unable to participate; patients with doubtful compliance; patients with inaccessibility to follow-up; patients who are unwilling or unable to give informed consent; and patients who are pregnant are all excluded.

Leg raise and leg fold physical maneuver technique

**Figure 1:**
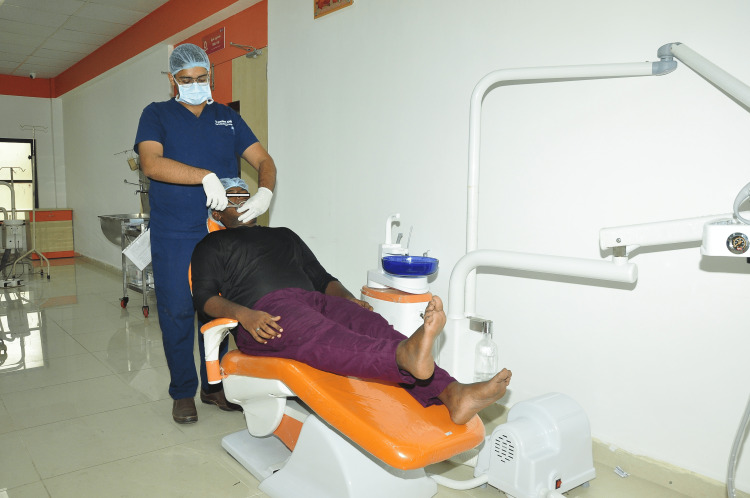
Patient performs leg raise maneuver in the dental chair during extraction

Group I (study group) patients undergoing extraction were educated few physical maneuvers that are to be done by the patients at regular intervals. Before extraction, the patients were educated about the physical maneuver that consists of leg lifts and leg folds. The clinician taught the patient to do this maneuver before local anesthesia administration, intraoperatively at regular intervals, if required postoperatively. The patient will raise his/her leg for five times to a minimum height of at least 15 cm (Figure 1) while sitting in the dental chair. Each leg is done individually or combined according to the patient's preference.

**Figure 2:**
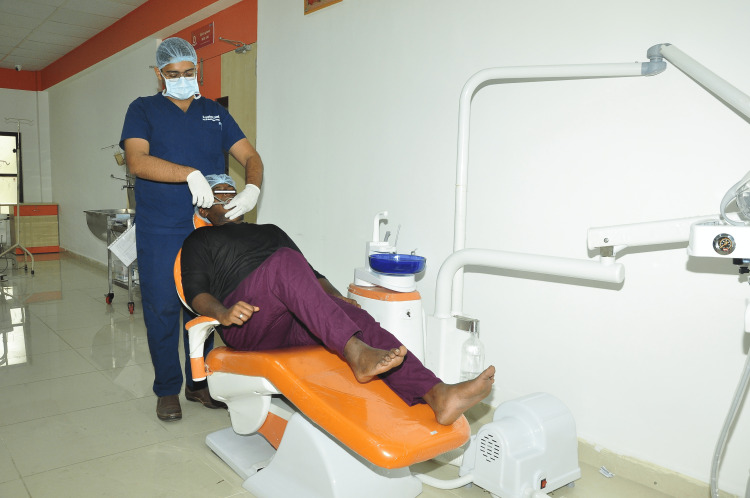
Patient performs leg fold maneuver in the dental chair during extraction, while the operator performs extraction without any hindrance

Once the leg raise maneuver is completed, the patient will fold the leg and stretch each leg five times using the knee joint (Figure 2). During the maneuver, sudden jerky movements should be avoided, and the patient is advised to focus primarily on physical activity.

Group II (Control group) patients conventionally underwent extraction. Informed consent was obtained from all the patients. The parameters for this study included monitoring blood pressure, SpO_2_, and pulse, which were monitored preoperatively, postoperatively, and verbally. Blood pressure was monitored using a manual sphygmomanometer. Pulse rate and SpO_2_ were assessed using a pulse oximeter. While extracting, if the patient develops any signs of unrest requiring emergency care, the procedure will be completely stopped, and maintenance of the chair in reverse trendelenburg and protocols for standard management of syncope will be followed.

## Results

Table 1 shows the baseline characteristics.

**Table 1:** Baseline characteristics DM: Diabetes mellitus; SHT: Subclinical hypothyroidism; CAD: Coronary artery disease; NRH: Nodular regenerative hyperplasia. * Statistically significant.

Variables	Categories	Control group, n (%)	Test group, n (%)	P-value
Gender	Males	9 (60)	8 (53.3)	0.713
	Females	6 (40)	7 (46.7)	
	Total	15 (100)	15 (100)	
Medical history	DHT, DM	0 (0)	1 (6.7)	1.000
	DM	1 (6.7)	1 (6.7)	
	DM, SHT	3 (20)	3 (20)	
	NRH	9 (60)	10 (66.7)	
	SHT	1 (6.7)	0 (0)	
	SHT + CAD	1 (6.7)	0 (0)	
	Total	15 (100)	15 (100)	
Previous history of syncope	Yes	15 (100)	15 (100)	-
	Total	15 (100)	15 (100)	
Previous history of extraction	No	5 (33.3)	3 (20)	0.682
	Yes	10 (66.7)	12 (80)	
	Total	15 (100)	15 (100)	
Age	£20 years	0 (0)	3 (20)	0.026*
	21-40 years	6 (40)	10 (66.7)	
	41-60 years	7 (46.7)	2 (13.3)	
	>60 years	2 (13.3)	0 (0)	
	Total	15 (100)	15 (100)	

Table 2 shows a comparison between the study and control groups using the chi-square test for the variables assessed. The occurrence of syncope, giddiness, and clammy skin was monitored. Statistically, a significant difference was observed between the groups in terms of syncope and giddiness experienced by patients (p < 0.05). This indicates that the physical maneuvering technique had a significant influence on the test group. No participant in the test group experienced post-treatment syncope, while five (33.3%) subjects in the control group experienced post-treatment syncope. Three participants in the test group experienced post-treatment giddiness, while nine participants in the control group experienced post-treatment giddiness. No significant difference was observed between the groups in terms of sweating and clammy skin.

**Table 2:** Comparison of the presence or absence of syncope and its features post-treatment between the study and control groups by Chi-square test * p-value less than 0.05 is statistically significant. n: Sample size.

Variables	Categories	Control group, n (%)	Test group, n (%)	Chi-square value	P-value
Syncope	No	10 (66.7)	15 (100)	6.000	0.014*
	Yes	5 (33.3)	0 (0)		
	Total	15 (100)	15 (100)		
Giddiness	No	6 (40)	12 (80)	5.000	0.025*
	Yes	9 (60)	3 (20)		
	Total	15 (100)	15 (100)		
Sweating	No	7 (46.7)	8 (53.3)	0.133	0.715
	Yes	8 (53.3)	7 (46.7)		
	Total	15 (100)	15 (100)		
Cold clammy skin	No	9 (60)	10 (66.7)	0.144	0.705
	Yes	6 (40)	5 (33.3)		
	Total	15 (100)	15 (100)		

Table 3 shows the comparison of systolic and diastolic blood pressure, oxygen saturation, and pulse rate pre-op and post-op in the test group using paired t-test. A significant difference was observed (p < 0.001) in terms of postoperative systolic blood pressure and pulse rate monitored. The mean systolic blood pressure was 116 ± 9.856 before treatment, which increased to 136 ± 8.281 after treatment. Similarly, the mean pulse rate pretreatment was observed to be 72.2 ± 3.167, which increased to 80.07 ± 4.728 post-treatment. Blood pressure and pulse rate seemed to increase in the control group; however, it was maintained within permissible limits. No significant difference was observed in the case of diastolic blood pressure and oxygen saturation.

**Table 3:** Comparison of systolic and diastolic blood pressure, oxygen saturation, and pulse rate preoperative and postoperative in the test group by paired t-test ** p-value less than 0.05 is statistically significant. Pre-op:-Pre-operative; post-op: Postoperative.

Variables		Mean	Standard deviation	Mean difference	95% confidence interval of mean difference		P-value
					Lower bound	Upper bound	
Systolic blood pressure	Pre-op	116.00	9.856	-20.00	-28.630	-11.370	<0.001**
	Post-op	136.00	8.281				
Diastolic blood pressure	Pre-op	75.33	7.432	-4.00	-8.080	0.080	0.054
	Post-op	79.33	7.037				
Oxygen saturation	Pre-op	100.27	1.335	0.267	-0.472	1.006	0.452
	Post-op	100.00	0.000				
Pulse rate	Pre-op	72.20	3.167	-7.867	-10.587	-5.147	<0.001**
	Post-op	80.07	4.728				

Table 4 shows the comparison of mean systolic and diastolic blood pressure, pulse rate, and oxygen saturation post-op between test and control groups using the independent samples t-test. A significant difference was observed between the groups in terms of both systolic and diastolic blood pressure as well as pulse rate (p < 0.001). The mean systolic blood pressure in the control group was 109.33 ± 8.837, while it was 136 ± 8.281 in the test group. The mean diastolic blood pressure was noted to be 69.33 ± 5.936 in the control group, while it was higher in the test group (79.33 ± 7.037). No difference was observed in the mean oxygen saturation levels between groups. The mean pulse rate was 68.93 ± 3.515 in the control group, while it was observed to be 80.07 ± 4.728 in the test group.

**Table 4:** Comparison of mean systolic and diastolic blood pressure, pulse rate, and oxygen saturation post-op between test and control groups by independent samples t-test ** p-value less than 0.05 is statistically significant.

	Groups	Mean	Standard deviation	Mean difference	95% confidence interval of mean difference		P-value
					Lower bound	Upper bound	
Systolic blood pressure	Control group	109.33	8.837	-26.67	-33.072	-20.261	<0.001**
	Test group	136.00	8.281				
Diastolic blood pressure	Control group	69.33	5.936	-10.00	-14.241	-8.018	<0.001**
	Test group	79.33	7.037				
Oxygen saturation	Control group	100.00	0.000	0.00	-	-	-
	Test group	100.00	0.000				
Pulse rate	Control group	68.93	3.515	-11.14	-14.869	-5.131	<0.001**
	Test group	80.07	4.728				

The above findings indicate that physical maneuvering has significantly influenced the blood pressure and pulse rate of test group subjects as compared to the control group.

## Discussion

The level of cerebral blood flow required for the maintenance of consciousness is 30 ml of blood per 100 g of brain tissue per minute. The adult brain weighs about 1360 g. Normal cerebral blood flow is 50-55 ml per 100 g per minute. During the minor surgical procedure, the patient is held in a semi-supine to an upright position, which impairs the heart’s ability to deliver blood to the brain and can cause syncope [20]. The diagnosis of vasovagal syncope was based on the definition of the guidelines of the European Society of Cardiology (ESC) [21]. Vasovagal syncope has a significant impact on the extraction of teeth, which leads to an uneventful medical emergency. Although many precautions are taken before extraction, there are no preventive techniques described in the literature to avoid syncope during extraction. Physical maneuvers, such as leg raise and leg fold, were used in this study as a preventive measure to avoid syncope during extraction. This study is based on earlier evidence that physical maneuvers reduce the incidence of syncope in patients with chronic vasovagal syncope. Alizadeh et al. in their study compared two physical maneuvers, hand grips and squatting, in a patient with a previous history of syncope. From their study, it was understood that the use of the two physical maneuvers in comparison to the control group significantly decreased the occurrence of vasovagal syncope in the long term [22]. Kim et al. also demonstrated that squatting with muscle tensing could increase blood pressure more effectively than hand grip, suggesting that it could be an effective technique for preventing vasovagal syncope, as it is more convenient and likely than the hand grip maneuver [23].

Brignole et al. showed in their study that isometric arm contraction, which is feasible, safe, and well-accepted by the patient, can abort impending vasovagal syncope by increasing systemic blood pressure [24]. Seals stated in a study that there is an influence of muscle mass on sympathetic neural activation during isometric exercise; the percentage of maximal voluntary force and the magnitude of the increase in blood pressure observed at any absolute point in time are directly related to the size of the contracting muscle mass. This augmented response is associated with a correspondingly greater increase in heart rate and thus appears to be mediated in part by a greater increase in cardiac output [25-30]. In our study, we used the same large muscles of the legs to increase cardiac output, which in turn increases cerebral perfusion and thus produces net results. Physical maneuvers can increase arterial pressure followed by their ability to increase peripheral resistance and sympathetic nerve activity in patients. This method proved to be highly effective. Prior counseling and time spent educating patients about this maneuver also aided with an improved patient relationship. None of the patients had any difficulty in executing the maneuver; however, repeated reminders to perform the leg raise and leg fold were required. Few patients had jerky movements while performing maneuvers, and they were instructed to slow down. It was mostly due to the anxiety of patients.

However, these jerky movements did not interrupt the procedure in most instances. If found to be highly disturbing, a momentary pause from executing the extraction procedure can help. This study, however, proves that syncope during extraction had been prevented by increasing the venous return to the heart by the muscular movement of the legs, which helped to increase the cardiac output. As patients were focused on the execution of these maneuvers, anxiety also seemed to be less, with all patients having positive feedback about the leg raise and leg fold movements.

## Conclusions

Physical counterpressure maneuvers are a risk-free, effective, and low-cost treatment method for patients with vasovagal syncope. Leg raise and leg fold maneuvers improved the patient's hemodynamics. Leg raise and leg fold maneuvers can be used as simple and effective preventive maneuvers in patients with vasovagal syncope and dental anxiety.

## References

[1] Haas DA (2006). Management of medical emergencies in the dental office: conditions in each country, the extent of treatment by the dentist. Anesth Prog.

[2] Sheldon RS, Grubb BP 2nd, Olshansky B (2015). 2015 heart rhythm society expert consensus statement on the diagnosis and treatment of postural tachycardia syndrome, inappropriate sinus tachycardia, and vasovagal syncope. Heart Rhythm.

[3] Edmondson HD, Gordon PH, Lloyd JM, Meeson JE, Whitehead FI (1978). Vasovagal episodes in the dental surgery. J Dent.

[4] Romme JJ, van Dijk N, Boer KR, Dekker LR, Stam J, Reitsma JB, Wieling W (2008). Influence of age and gender on the occurrence and presentation of reflex syncope. Clin Auton Res.

[5] Alboni P, Messop AC, Lauri A, Furlan R (2021). Are women really more affected by vasovagal syncope than men?. J Cardiovasc Med (Hagerstown).

[6] Ganzeboom KS, Mairuhu G, Reitsma JB, Linzer M, Wieling W, van Dijk N (2006). Lifetime cumulative incidence of syncope in the general population: a study of 549 Dutch subjects aged 35-60 years. J Cardiovasc Electrophysiol.

[7] Park J, Jang SY, Yim HR (2010). Gender difference in patients with recurrent neurally mediated syncope. Yonsei Med J.

[8] Lee YH, Cha SI, Shin KM (2018). Clinical relevance of syncope in patients with pulmonary embolism. Thromb Res.

[9] Grossman SA, Shapiro NI, Van Epp S (2005). Sex differences in the emergency department evaluation of elderly patients with syncope. J Gerontol A Biol Sci Med Sci.

[10] Deveau AP, Sheldon R, Maxey C, Ritchie D, Doucette S, Parkash R (2020). Sex differences in vasovagal syncope: a post hoc analysis of the prevention of syncope trials (POST) I and II. Can J Cardiol.

[11] Duncan GW, Tan MP, Newton JL, Reeve P, Parry SW (2010). Vasovagal syncope in the older person: differences in presentation between older and younger patients. Age Ageing.

[12] Marks LA, Van Parys C, Coppens M, Herregods L (2013). Awareness of dental practitioners to cope with a medical emergency: a survey in Belgium. Int Dent J.

[13] Atherton GJ, McCaul JA, Williams SA (1999). Medical emergencies in general dental practice in Great Britain. Part 1: their prevalence over a 10-year period. Br Dent J.

[14] Atherton GJ, McCaul JA, Williams SA (1999). Medical emergencies in general dental practice in Great Britain. Part 2: drugs and equipment possessed by GDPs and used in the management of emergencies. Br Dent J.

[15] Shim YS, Kim AH, Jeon EY, An SY (2015). Dental fear & anxiety and dental pain in children and adolescents; a systemic review. J Dent Anesth Pain Med.

[16] Armfield JM, Spencer AJ, Stewart JF (2006). Dental fear in Australia: who's afraid of the dentist?. Aust Dent J.

[17] Nicolas E, Collado V, Faulks D, Bullier B, Hennequin M (2007). A national cross-sectional survey of dental anxiety in the French adult population. BMC Oral Health.

[18] Armfield JM, Heaton LJ (2013). Management of fear and anxiety in the dental clinic: a review. Aust Dent J.

[19] Malamed SF (1997). Emergency medicine: beyond the basics. J Am Dent Assoc.

[20] Malamed SF, Orr DL (2014). Medical Emergencies in the Dental Office.

[21] Moya A, Sutton R, Ammirati F (2009). Guidelines for the diagnosis and management of syncope (version 2009). Eur Heart J.

[22] Alizadeh A, Peighambari M, Keikhavani A, Emkanjoo Z, Assadian M (2016). The role of acute physical maneuver in preventing vasovagal syncope: a randomized clinical trial. Age.

[23] Kim KH, Cho JG, Lee KO (2005). Usefulness of physical maneuvers for prevention of vasovagal syncope. Circ J.

[24] Brignole M, Croci F, Menozzi C (2002). Isometric arm counter-pressure maneuvers to abort impending vasovagal syncope. J Am Coll Cardiol.

[25] Seals DR (1989). Influence of muscle mass on sympathetic neural activation during isometric exercise. J Appl Physiol.

[26] Buck JA, Amundsen LR, Nielsen DH (1980). Systolic blood pressure responses during isometric contractions of large and small muscle groups. Med Sci Sports Exerc.

[27] Coote JH, Hilton SM, Perez-Gonzalez JF (1971). The reflex nature of the pressor response to muscular exercise. J Physiol.

[28] Seals DR, Washburn RA, Hanson PG, Painter PL, Nagle FJ (1983). Increased cardiovascular response to static contraction of larger muscle groups. J Appl Physiol Respir Environ Exerc Physiol.

[29] Freyschuss U (1970). Elicitation of heart rate and blood pressure increase on muscle contraction. J Appl Physiol.

[30] Rusch NJ, Shepherd JT, Webb RC, Vanhoutte PM (1981). Different behavior of the resistance vessels of the human calf and forearm during contralateral isometric exercise, mental stress, and abnormal respiratory movements. Circ Res.

